# Testing the Energy-Environmental Kuznets Curve Hypothesis in the Renewable and Nonrenewable Energy Consumption Models in Egypt

**DOI:** 10.3390/ijerph18147334

**Published:** 2021-07-08

**Authors:** Haider Mahmood, Tarek Tawfik Yousef Alkhateeb, Muhammad Tanveer, Doaa H. I. Mahmoud

**Affiliations:** 1 Department of Finance, College of Business Administration, Prince Sattam bin Abdulaziz University, Alkharj 11942, Saudi Arabia; 2 Department of Agricultural Economics, Kafrelsheikh University, Kafrelsheikh 33511, Egypt; tkhteb1@gmail.com; 3 Prince Sultan University, Rafha Street, Riyadh 11586, Saudi Arabia; mtanveer@psu.edu.sa; 4 Economics and Agribusiness Department, Faculty of Agriculture, Alexandria University, Alexandria 21545, Egypt; doaa.mahmoud@alexu.edu.eg

**Keywords:** energy-EKC, economic growth, primary energy consumption, oil, coal, and natural gas consumption, hydroelectricity consumption

## Abstract

The Environmental Kuznets Curve (EKC) association between income and pollution emissions proxies has been extensively tested in the environmental literature. However, testing of the energy-EKC is scanty. This present research examined the energy–EKC in the cubic relationship of economic growth and different renewable and nonrenewable energy proxies in Egypt from 1965–2019. In the long run, we corroborate the N-shaped relationships in the case of primary energy, oil, and coal consumption models, and confirm the long run energy–EKC association in these energy proxies. Moreover, we find turning points of the N-curve for these energy sources in 1998, 2000, and 1979–2005, in primary energy, oil, and coal consumption models, respectively. Hence, economic growth is responsible for increasing nonrenewable energy consumption and has environmental consequences in Egypt. In the short run, we find N-shaped relationships in the case of primary energy, oil, and coal consumption. Further, we find an inverted U-shaped relationship in the case of natural gas consumption. In addition, we corroborate an inverted N-shaped relationship in the case of hydroelectricity consumption, a renewable energy source. Hence, we confirm the short-run energy–EKC relationship in all investigated renewable and nonrenewable energy proxies.

## 1. Introduction

**Figure 1:**
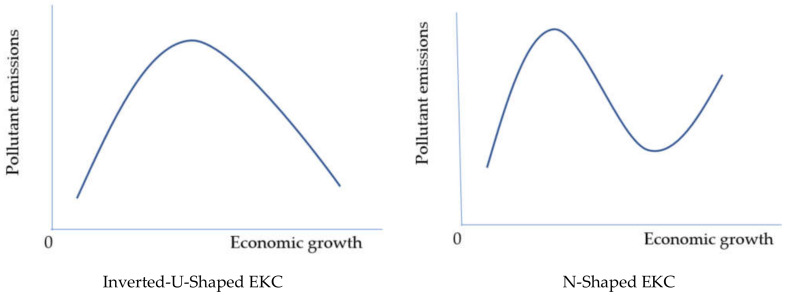
The EKC.

Economic growth raises energy consumption and pollution emissions during the first phase of development, called the ‘scale effect’. Later, it may also reduce the quantity or type of energy demand and reduce pollution emissions. This can be due to the technique effect, where the latest clean technology may reduce energy demand and pollution. Moreover, pollution-oriented production would tend to be replaced by clean products, and the composition effect would help to reduce pollution emissions [1]. The phenomenon of increasing pollution at the first stage and decreasing at the second stage is termed the environmental Kuznets curve (EKC) hypothesis [2], based on Kuznets [3]. This reflects an inverted U-shaped association between economic growth and pollution, shown in Figure 1. Therefore, the quadratic effect of income growth on pollution may be tested to verify the EKC hypothesis. Moreover, another stream of the EKC literature has tested the cubic impact of income on pollution emissions, which portrays a positive effect of growth in the first stage, a negative impact in the second stage, and a positive impact in the third stage of development. This phenomenon is the N-shaped EKC [4], shown in Figure 1.

The literature has probed the above types of EKC using different proxies for pollution emissions, various determinants of pollution emissions along with economic growth, and different methodologies. In empirical testing, the relationship of economic growth with emissions in the form of carbon, sulfur, nitrogen, greenhouse gases (GHGs), and the ecological footprint is varied. This can be a monotonic, inverted U-curve, U-curve, N-curve, inverted N-curve, or no relation. The monotonic nature reveals the linear impact of economic growth on any form of emission, which can be increasing or decreasing, while the squared and cubic terms of income should appear insignificant. A significant increase in emissions accompanied by a significant initial fall in emissions due to a substantial increase in income provides us with evidence of an inverted U-curved relation. If emissions again later increase, this would be defined as an N-curve relationship [5]. Opposite cases have also been reported in the literature, designated as a U-curve [6] and inverted N-curve between growth and emissions [7]. In the relationships discussed above, an inverted U-curve and N-curve may be claimed in order to validate the EKC hypothesis [8,9].

The literature has also inspected the EKC between energy use and economic growth, termed energy–EKC (EEKC). This EEKC testing is more relevant in countries that depend on nonrenewable energy consumption intensively. The literature has investigated the EEKC in the quadratic and cubic relationship between energy and growth. For instance, in a panel study, Suri and Chapman [10] initiated empirical testing of the EEKC. They argued that manufactured exports, imports, and gross domestic product (GDP) significantly affected energy consumption and economic growth, because manufactured exports and imports changed the structure of the economy in terms of production. For instance, manufacturing imports may reduce local production and energy consumption. On the other hand, manufacturing exports may increase local production and energy consumption. Hence, manufacturing imports may reduce the manufacturing GDP, manufacturing exports may increase the manufacturing GDP, and both might shape the economic structure in terms of manufacturing. Empirical testing corroborated the inverted U-curve between GDP and energy use, hence validating the EEKC hypothesis. Moreover, the positive effects of manufacturing GDP and exports and the negative effect of manufacturing imports were found on energy consumption. Thus, the manufacturing structure of the economy did matter regarding energy consumption. Aruga [11] investigated the EEKC in 19 Asian countries regarding the quadratic relationship of GDP and energy consumption. The EEKC was corroborated in high-income Asian countries, while EEKC was not substantiated in middle-income economies. Hence, energy transition was only found in the case of high-income Asian countries.

In country-specific studies, Hundie and Daksa [12] investigated the EEKC in the quadratic settings between growth and energy intensity in Ethiopia from 1974–2014. They validated the EEKC and found positive effects of urbanization and foreign aid on energy intensity. However, the impact of imports was negative, and the effect of industrial GDP on energy intensity was insignificant. Aboagye [13] investigated the quadratic relationship between energy variables and growth in Ghana from 1981–2014. The EEKC was validated in both models of energy consumption and intensity. Moreover, urbanization, industrial activities, and trade openness raised energy consumption, while Foreign Direct Investment (FDI) helped to reduce it. On the other hand, industrial activities and FDI had positive effects on energy intensity.

Later, the literature started to probe the cubic relationship between growth and energy variables. For instance, Luzzati and Orsini [14] explored the N-curve between absolute energy use and income in 113 countries from 1971–2004. They could not validate the EEKC in empirical testing of the whole panel. However, they found positive effects of income and income-cubic terms and an insignificant impact of income-square on energy use in 36 low-income countries. The positive and negative effects of income-square and income-cubic terms were found on energy use in 38 middle-income and nine oil-producing countries. In contrast, the effect of the income-linear term was insignificant. Moreover, the positive and negative effects of income and income-cubic terms were found on energy use in 25 high-income countries. In contrast, the impact of the income-square term was insignificant. Hence, evidence for EEKC was not validated in any investigated group of countries. Pablo-Romero and De Jesus [15] examined EEKC in 22 American economies during 1990–2011. They found positive effects of linear, squared, and cubic terms of gross value added to absolute energy consumption. Hence, EEKC was not validated in the investigated region.

**Figure 2:**
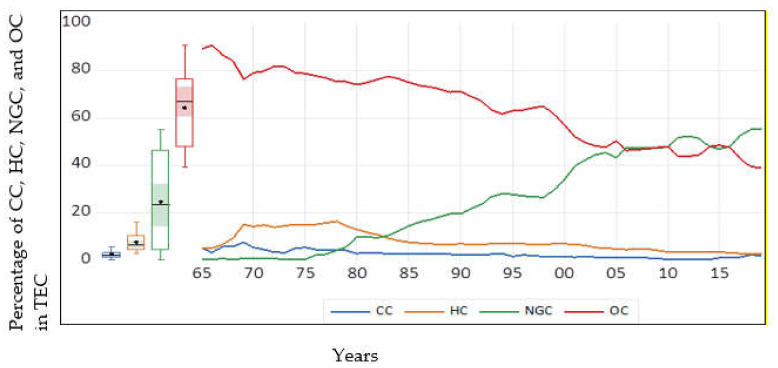
Trends of Energy Sources.

Egypt is a significant energy consumer and the leading producer of oil and gas in Africa. Figure 2 shows the percentage of different sources of energy consumption, i.e., oil consumption (OC), natural gas consumption (NGC), coal consumption (CC), and hydroelectricity consumption (HC). The data on all energy sources are taken from BP [16], expressed in exajoules and converted into a percentage of total energy consumption (TEC). The boxplot of OC shows that minimum, median and maximum values are 39.28%, 67.18%, and 90.85% of OC in TEC. The maximum value is observed in 1966. Afterward, oil consumption declines but accounts for more than 50% of TEC till 2001 and around 40% in 2018–19. In the same way, coal consumption has an average negative trend in the sample period, after touching a peak in 1969. The boxplot of CC shows that minimum, median and maximum values are 0.47%, 2.42%, and 7.59% of CC in TEC. Natural gas consumption has the opposite trend compared to oil and coal consumption. The average trend of NGC is positive in the sample period, significantly jumped after 1998. The minimum, median and maximum values are 0.39%, 23.67%, and 55.43% of NGC in TEC. Natural gas consumption generally emits less CO_2_ emissions than oil and coal consumption [17]. Hydroelectricity consumption has an average positive trend during 1965–1975 but has a mostly negative trend afterward. The minimum, median and maximum values are 3.08%, 6.90%, and 16.36% of HC in TEC. In summary of Figure 2, we may observe that Egypt fulfils more than 90% of energy demand from nonrenewable sources in most sample periods except 1969–1982. Further, Egypt has also been a net importer of oil since 2009 [18]. Hence, energy consumption sources in Egypt may have environmental consequences, and investigation of these energy sources is important during the process of the country’s economic growth.

Increasing economic growth is responsible for accelerated energy consumption [19,20]. In this situation, it seems pertinent to conduct a comprehensive study to investigate the effect of different phases of economic growth on energy consumption, which can be tested with the cubic EEKC hypothesis. Particularly, Figure 2 shows the importance of EEKC analyses of different energy sources in Egypt, because all energy sources have different trends. In the global literature, the EEKC has been investigated considering a quadratic relationship to energy and growth [10,11,12,13] and considering a cubic relationship to energy and growth [14,15,20,21,22]. In the case of Egypt, the literature has investigated the linear impact of income on energy consumption, ignoring these quadratic or cubic relationships [19,20,23,24]. Hence, the testing of EEKC in the relationship between growth and energy is absent in the Egyptian literature. However, some studies have investigated the EKC between economic growth and emissions, considering a quadratic relationship [25,26,27,28,29,30,31]. Therefore, the Egyptian literature omits testing of the EKC in its cubic relationship to economic growth and emissions and has ignored the EEKC in both quadratic and cubic relationships of economic growth and energy variables. In the present state of the art, this study aims to test the EEKC in the cubic relationship of economic growth and energy consumption to fill this literature gap. To ensure a comprehensive empirical contribution, the EEKC is tested in disaggregated renewable and nonrenewable energy sources, using a maximum time sample of 1965–2019.

The rest of the study is ordered as follows: a thorough discussion is carried out on the empirical findings of various scholars in Section 2. In Section 3, sources of data, models, and econometric methods are discussed. Section 4 provides details of the empirical findings. The last and final section sheds light via concluding remarks.

## 2. Literature Review

Grossman and Krueger [1] initiated the testing of the EKC between economic growth and different proxies of emissions. They investigated the role of the North American Free Trade Agreement (NAFTA) on pollution emissions. They found that trade reduced emissions in high-income economies and increased emissions in low-income economies. Hence, the level of development and trade are responsible for the environmental quality of an economy. Increasing exports would increase the size of the economy and could pollute the environment through the scale effect in the first stage of development. Later, with increasing levels of development, trade might shift pollution-oriented production from a rich economy (with strong environmental regulations because of a demand for a cleaner environment) to a poor economy (with weak environmental regulations) [32], termed as the Pollution Haven Hypothesis (PHH). Heckscher-Ohlin’s (H-O) theory also explained that free trade promoted labor and natural resource-intensive activities in developing countries and human capital-intensive activities in developed countries. Hence, such specialization might shift pollution from high to middle-income countries [33]. In this context, Saint-Paul [34] argued that poor economies tend to be a net exporter of pollution-oriented products and vice versa in the case of rich economies. Hence, pollution-oriented industry would be displaced from developed to developing countries, changing the composition of these economies [35]. Thus, trade would shift developed economies from the first to the second stage of the EKC. The second stage of the EKC starts once the technique and composition effects dominate the scale effect within the economy [36]. Moreover, trade would also help developing economies to shape their EKC. For instance, trade could improve the economic growth of developing economies, which might demand tight environmental policies due to the demand for a cleaner environment [37].

Arrow et al. [38] argued that the EKC theory assumed economic growth as an exogenous variable. Hence, the EKC considered only a one-way relationship, from economic activities to emissions. However, it ignored the possible feedback effect between environment and economic growth because environmental damage might also reduce economic growth due to demand for tight environmental policies. In a piece of empirical evidence, Coondoo and Dinda [39] found a feedback effect between CO_2_ emissions and economic growth in developed economies. However, this feedback effect was not found in developing economies. Hence, any environmentally sustainable policy might reduce economic activities in a developed economy. Moreover, the environmental policy might also shift pollution activities from developed to developing countries [40]. In addition, Stern [41] provided some econometric criticism of the EKC estimation, i.e., simultaneity, heteroskedasticity, cointegration issues, and omitted variables bias. These econometric problems were mostly suggested in panel models’ estimations, ignoring time or cross-sectional effects. Moreover, Stern and Common [42] pointed out that time or cross-sectional effects could be correlated with income level and square variables. Stern [41] suggested the decomposition and frontier model approach using linear programming, assuming best-practice technologies on the frontier. Frontier models did not need sectoral fuel consumption data. In the same way, Millimet et al. [43] proposed the semi-parametric approach to reduce the peaks of the inverted U-shaped relationship. Grossman and Krueger [36] proposed a decomposition approach to study emissions, from GDP, emissions intensity, and sectoral share in GDP, which was later augmented by including fossil and non-fossil fuel.

Ignoring EKC, the literature has investigated the relationship between energy consumption and economic growth via causality analysis and reported different conclusions. For example, Akkemike and Göksal [44] found a two-way relationship between GDP and energy consumption and corroborated the feedback hypothesis in most of their investigated countries. On the other hand, Payne [45] could not validate any direction of the relationship between energy consumption and economic growth and confirmed the neutrality hypothesis. Moreover, Ouedraogo [46] reported the one-way long-run relationship between energy consumption and economic growth and corroborated the growth hypothesis in the long run. However, one-way short-run causality was found from GDP to energy consumption, and the conservative hypothesis was verified in the short run. Further, Huang et al. [47] found a one-way relationship between economic growth and energy consumption high and middle-income countries and confirmed the conservative hypothesis.

Some literature has also considered both the EKC testing and causality analyses. For example, Shahbaz et al. [48] scrutinized the EKC in Pakistan. This study used a causality test from 1971 to 2009 and confirmed a one-way causation from real income and its square to pollution in the short run. Nasir and Rehman [49] examined the relation between GDP and emissions in Pakistan. After using Johansen’s multivariate cointegration and causality test for the data from 1972 to 2008, this study observed that GDP enhanced pollution and GDP-squared reduced pollution. Hence, the EKC was corroborated, and the authors also provided evidence of one-way causality running from the GDP and GDP-squared to pollution. Shahbaz et al. [50] tested the EKC for an Indian economy using Bayer and Hanck [51] and ARDL for the sample period from 1970 to 2012. The authors found that increasing growth accelerated emissions. Once income had increased beyond a certain threshold, emissions started to decrease. Hence, the results confirmed the EKC and also reported two-way causality between growth and emissions. Jalil and Mahmud [52] conducted research on testing EKC in China. Using ARDL bounds testing from 1975 to 2005, the study confirmed evidence of EKC along with the quadratic effect of real income on CO_2_ emissions in China. The study also provided evidence of a causal relationship between real income and its square and emissions.

Shahbaz et al. [53] considered the nonlinear impact of financial development on emissions in the EKC framework in the UAE. After employing ARDL for the data series from 1975-Q1 to 2014-Q4, the study could not find the EKC in UAE. The study further reported a two-way causal link between both actors. Ozturk and Acaravci [54] explored the EKC in Turkey. After considering the bounds testing approach from 1960 to 2007, the results exposed evidence of the EKC between GDP and emissions. The results further revealed the long-run unidirectional causal relation between GDP and emissions in Turkey. Shahbaz et al. [55] examined the nonlinear impact of real GDP on emissions in Turkey. Using a sample from 1970 to 2010, the authors reported a positive effect of GDP and a negative effect of GDP-squared on emissions. Further, the results displayed a short-run causality between GDP and its square and emissions. In the long run, bidirectional causality between both variables was seen in Turkey. Ozatac et al. [56] explored the EKC in Turkey. After employing the ARDL approach on the data series from 1960 to 2013, the results corroborated the EKC in Turkey. The study also reported one-way causality between growth and emissions.

A vast literature has examined the EKC, ignoring the possible feedback effect between energy and economic growth variables [38]. For instance, Acaravci and Ozturk [57] conducted research on 19 European countries to explore the EKC. The study used the Autoregressive Distributive Lag (ARDL) model from 1960 to 2005 and confirmed the EKC in Denmark and Italy. Madaleno and Moutinho [58] studied the EKC between GHGs and the income of 27 European Union countries. The authors found a U-curve between emission and growth relationships in the models of different GHG emissions in the short run. Moreover, electricity and total energy use had positive effects on most of the investigated emissions. To et al. [59] examined the EKC in emerging Asian countries during 1980–2016. The authors found an inverted N-curve between income and emissions and an inverted U-curve between FDI and emissions.

Javid and Sharif [60] inspected the nonlinear impact of economic growth on emissions. They used the bounds test for the sample from 1972 to 2013 in Pakistan. They found that pollution emissions increased due to an increase in economic growth up to a point. However, beyond this level, it started to decrease as economic growth increased. Hence, they confirmed the presence of the EKC in Pakistan. Murshed et al. [61] tested the nonlinear impact of GDP on GHGs by incorporating control variables such as energy intensity, liquefied petroleum gas, coal, natural gas, oil, and hydropower consumption in Bangladesh. After employing a bound test for a sample from 1980 to 2015, this study provided evidence of the EKC between GDP and GHGs. In the same way, Rabbi et al. [62] inspected the EKC in Bangladesh. The study used Johansen’s multivariate approach for the data series from 1972 to 2012 and found a long-run EKC and short-run U-shaped relationship.

Dong et al. [63] examined the EKC in China. The study confirmed cointegration between the emissions and their determinants in China from 1993 to 2016. The long-run results concluded the EKC between real GDP and emissions in China. Ren et al. [64] took 18 industries in China and tested the EKC. Using a sample from 2000 to 2010, the empirical results supported the EKC hypothesis in all selected 18 industries. Farhani and Lorente [65] examined the nonlinear impact of GDP on emissions by taking three control variables in three separate specifications. Using a sample from 1965 to 2017, the empirical results reported a U-shaped relationship using coal and oil consumption as control variables in China. Results also supported the EKC hypothesis in China, controlling gas consumption. The results further disclosed the EKC hypothesis in the USA, controlling coal, gas, and oil consumption. Lastly, no evidence of EKC was found regarding the control of coal and oil consumption in India. However, the EKC was found, controlling gas consumption, in the Indian model.

Saboori et al. [66] explored the EKC in Malaysia. The study used the ARDL approach on the sample period from 1980 to 2008 and corroborated the EKC between emissions and income in Malaysia. Saboori and Sulaiman [67] examined the EKC between income and emissions. The study used the ARDL cointegration approach by using four controls in alternate functional forms, such as coal, gas, oil, and electricity consumption, on the data series from 1980 to 2009 and found evidence of the EKC in Malaysia. Marques et al. [68] inspected the persistence of EKC in Australia. The study considered the bound test for the sample from 1965 to 2016 and witnessed the long-run EKC hypothesis. The U-formed relation was evident in the short run, which established an absence of the short-run EKC in Australia. Pata [69] conducted research testing the EKC between real income and emissions in Turkey. The results provided evidence of the EKC in Turkey for a sample from 1971 to 2014.

Bilgili et al. [70] scrutinized the EEKC between growth and energy intensity in Asia. After employing an augmented mean group estimator on the sample from 1990 to 2014, the results provided evidence of the EEKC in Malaysia, India, Nepal, China, Indonesia, Vietnam, and the Philippines. However, the EEKC was not evident in Thailand, Bangladesh, and South Korea. In Panel, the study further provided evidence of one-way causation from growth to energy intensity. Kander et al. [71] considered consumption-based energy rather than a production-based approach in order to test the relevance of the EEKC in seven European economies. After using a sample from 1870 to 1935, the study found evidence of the EEKC between income and production-based energy intensity in Germany and the Czech Republic. The study further reported a slight bottom-up U-curve in Portugal but a flat long-run bottom-up U-curve in the case of Great Britain. The study exposed that the EKC was not evident in Italy, Denmark, and Sweden and found evidence of EEKC between growth and consumption-based energy intensity in Portugal. A slight bottom-up U-curve was prominent in Germany, and EEKC between income and consumption-based energy intensity was not found in Denmark, Italy, Sweden, and Great Britain. It concluded that the EEKC was relevant to production-based energy intensity, while it became weak or almost irrelevant in the case of consumption-based energy.

Considering spatial autocorrelation, Li et al. [21] investigated the 21 regions of China from 2000–2017 to test the cubic relationship between growth and different energy sources, controlling urbanization, industrialization, and population density. They corroborated the inverted N-shaped relationship in oil, gas, coal, and total energy consumption models. Moreover, the positive effects of industrialization and population density on energy variables were found. However, the negative impact of urbanization was seen. In the same way, Ge et al. [22] investigated the impacts of income and urbanization on nitrogen oxide in the Chinese regions, considering spatial autocorrelation. The authors corroborated the inverted N-shaped relationships between emissions and income and between urbanization and emissions. Moreover, energy intensity accelerated these emissions.

Ignoring the EEKC, the literature has investigated the EKC between growth and emission relationships in Egypt. For instance, Ibrahiem [25] studied the EKC between CO_2_ emissions and income during 1980–2010. The EKC was not validated in the empirical exercise. Moreover, energy consumption positively affected emissions, and population and trade openness negatively affected emissions. Moreover, bidirectional causality was found between emissions and growth. Mahmood et al. [26] probed the EKC in Egypt during 1990–2014, controlling trade, FDI, and energy use. Energy use showed a positive impact on emissions, and FDI negatively affected emissions, while trade showed an insignificant effect. Moreover, the EKC was found valid between emissions and growth. El-Aasar and Hanafy [27] examined the EKC between GHGs and growth in Egypt during 1971–2012, controlling trade. The EKC was not validated, and trade showed an insignificant effect on GHGs.

Onafowora and Owoye [72] investigated the EKC in eight countries’ time-series analyses, including Egypt. They corroborated the quadratic EKC in Japan and South Korea and the N-shaped EKC in other countries. Moreover, a positive effect of population density on emissions was reported in five out of eight investigated countries. Beşe and Kalayci [28] examined the EKC in Egypt, Turkey, and Kenya from 1971–2014. The EKC was not validated in all investigated countries. However, energy consumption caused the growth in Egypt and Kenya. Moosa [29] investigated the EKC in the quadratic effect of income on CO_2_ emissions in Jordon, Tunisia, Algeria, and Egypt during 1960–2014. The study found the EKC in all investigated countries. Using data from 1980–2014, Ahmed et al. [73] investigated the EKC in eight developing countries, including Egypt. The estimations corroborated the EKC in the panel and five out of eight counties’ time series results. However, the EKC was not validated in Egypt, Indonesia, and Malaysia. Moreover, imports and energy usage had positive effects on emissions.

Sghaier et al. [30] investigated the EKC in Tunisia, Morocco, and Egypt during 1980–2014, controlling tourism. They corroborated the EKC in Morocco and Egypt and a U-shaped relationship in Tunisia. Tourism showed a positive impact on emissions in Tunisia and Morocco and showed a negative effect in Egypt. Sulaiman and Abdul-Rahim [74] investigated the EKC in eight African countries’ panel, including Egypt, during 1980–2015, controlling biomass energy in analysis. They found the validity of EKC. Moreover, biomass energy usage helped to reduce CO_2_ emissions. Fethi and Senyucel [31] examined the EKC’s control of tourism in analyses of 50 tourist countries. The authors reported the EKC in the panel and most of the investigated countries’ time-series analyses. However, the EKC was not validated in Egypt. Moreover, energy consumption had positive and tourism had insignificant impact on emissions. However, both energy use and tourism accelerated emissions in Egypt.

Ignoring the EKC, some literature also investigated the linear relationship between energy and growth in Egypt. For instance, Abdel-Khalek [19] studied and found a positive effect of income on energy consumption, but of a low magnitude. Sharaf [20] performed a causality analysis and found causation between income and oil and electricity usage in Egypt. In addition, the literature has investigated the income and electricity consumption relationship and found that economic growth positively affected electricity consumption in Egypt [23,24].

This literature review has found many studies on EKC testing, but limited studies have investigated the EEKC, which has been tested hypothesizing an inverted U-shaped relationship between energy variables and economic growth [10,11,12,13]. Some studies have also considered the possible N-shaped relationship between energy variables and economic growth [14,15,20,21,22]. In the context of Egypt, literature has investigated the EKC in a relationship between pollutant emissions and economic growth [25,26,27,28,29,30,31,72,73]. However, the testing of the EEKC is absent in Egyptian literature, which motivates us to investigate N-shaped EEKC in the relationship between different sources of energy consumption and economic growth.

## 3. Methods

Following Luzzati and Orsini [14] and Pablo-Romero and De Jesus [15], we test the isolated N-shaped relationship between absolute energy consumption and GDP per capita in Egypt to verify the EEKC in the following manner:

EC_t_ = f (GDPC_t_, GDPC_t_^2^, GDPC_t_^3^) (1)

EC_t_ is the natural logarithm of energy consumption in exajoules, and GDPC_t_ is the natural logarithm of GDP per capita in constant Egyptian Pounds. GDPC_t_^2^ and GDPC_t_^3^ are square and cubic terms of GDPC_t_ to test the N-shaped EEKC. *t* is annual time series from 1965 to 2019. Li et al. [21] suggested an inquiry into the different proxies of disaggregated energy consumption in the testing of N-shaped EEKC. Therefore, we test the EEKC with the leading energy sources of Egypt in the following manner:

PEC_t_ = f (GDPC_t_, GDPC_t_^2^, GDPC_t_^3^) (2)

OC_t_ = f (GDPC_t_, GDPC_t_^2^, GDPC_t_^3^) (3)

NGC_t_ = f (GDPC_t_, GDPC_t_^2^, GDPC_t_^3^) (4)

CC_t_ = f (GDPC_t_, GDPC_t_^2^, GDPC_t_^3^) (5)

HC_t_ = f (GDPC_t_, GDPC_t_^2^, GDPC_t_^3^) (6)

PEC_t_, OC_t_, NGC_t_, CC_t_, and HC_t_ are natural logarithms of primary energy, oil, natural gas, coal consumption, and hydroelectricity consumption in exajoules. The data on five proxies of energy consumption are collected from BP [16], and GDP per capita is collected from the World Bank [75].

Before moving to the cointegration analyses, the testing of unit root is a prerequisite. First, we apply the Ng and Perron [76] test, due to its efficiency, in the small-time sample. Then the ARDL of Pesaran et al. [77] is utilized to confirm the long and short-run effects. The models in Equations (2)–(6) are expressed in the ARDL form as follows:

(7) $$∆{\mathrm{PEC}}_{t}=a_{0}+a_{11}{\mathrm{PEC}}_{t-1}+a_{12}{\mathrm{GDPC}}_{t-1}+a_{13}{\mathrm{GDPC}}_{t-1}^{2}+a_{14}{\mathrm{GDPC}}_{t-1}^{3}+\sum_{i=1}^{k1}a_{21i}∆{\mathrm{PEC}}_{t-i}\;+\sum_{i=0}^{k2}a_{22i}∆{\mathrm{GDPC}}_{t-i}+\sum_{i=0}^{k3}a_{23i}∆{\mathrm{GDPC}}_{t-i}^{2}+\sum_{i=0}^{k4}a_{24i}∆{\mathrm{GDPC}}_{t-i}^{3}+\omega_{1t}$$

(8) $$∆{\mathrm{OC}}_{t}=b_{0}+b_{11}{\mathrm{OC}}_{t-1}+b_{12}{\mathrm{GDPC}}_{t-1}+b_{13}{\mathrm{GDPC}}_{t-1}^{2}+b_{14}{\mathrm{GDPC}}_{t-1}^{3}+\sum_{i=1}^{l1}b_{21i}∆{\mathrm{OC}}_{t-i}\;+\sum_{i=0}^{l2}b_{22i}∆{\mathrm{GDPC}}_{t-i}\;+\sum_{i=0}^{l3}b_{23i}∆{\mathrm{GDPC}}_{t-i}^{2}+\sum_{i=0}^{l4}b_{24i}∆{\mathrm{GDPC}}_{t-i}^{3}+\omega_{2t}$$

(9) $$∆{\mathrm{NGC}}_{t}=c_{0}+c_{11}{\mathrm{NGC}}_{t-1}+c_{12}{\mathrm{GDPC}}_{t-1}+c_{13}{\mathrm{GDPC}}_{t-1}^{2}+c_{14}{\mathrm{GDPC}}_{t-1}^{3}+\sum_{i=1}^{m1}c_{21i}∆{\mathrm{NGC}}_{t-i}\;+\sum_{i=0}^{m2}c_{22i}∆{\mathrm{GDPC}}_{t-i}+\sum_{i=0}^{m3}c_{23i}∆{\mathrm{GDPC}}_{t-i}^{2}+\sum_{i=0}^{m4}c_{24i}∆{\mathrm{GDPC}}_{t-i}^{3}+\omega_{3t}$$

(10) $$∆{\mathrm{CC}}_{t}=d_{0}+d_{11}{\mathrm{CC}}_{t-1}+d_{12}{\mathrm{GDPC}}_{t-1}+d_{13}{\mathrm{GDPC}}_{t-1}^{2}+d_{14}{\mathrm{GDPC}}_{t-1}^{3}+\sum_{i=1}^{n1}d_{21i}∆{\mathrm{CC}}_{t-i}+\sum_{i=0}^{n2}d_{22i}∆{\mathrm{GDPC}}_{t-i}\;+\sum_{i=0}^{n3}d_{23i}∆{\mathrm{GDPC}}_{t-i}^{2}+\sum_{i=0}^{n3}d_{24i}∆{\mathrm{GDPC}}_{t-i}^{3}+\omega_{4t}$$

(11) $$∆{\mathrm{HC}}_{t}=e_{0}+e_{11}{\mathrm{HC}}_{t-1}+e_{12}{\mathrm{GDPC}}_{t-1}+e_{13}{\mathrm{GDPC}}_{t-1}^{2}+e_{14}{\mathrm{GDPC}}_{t-1}^{3}+\sum_{i=1}^{o1}e_{21i}∆{\mathrm{HC}}_{t-i}+\sum_{i=0}^{o2}e_{22i}∆{\mathrm{GDPC}}_{t-i}\;+\sum_{i=0}^{o3}e_{23i}∆{\mathrm{GDPC}}_{t-i}^{2}+\sum_{i=0}^{o4}e_{24i}∆{\mathrm{GDPC}}_{t-i}^{3}+\omega_{5t}$$

At first, we test the cointegration in Equations (7)–(11) of ARDL models using the bound test. The F-values of Kripfganz and Schneider [78] are utilized, due to their efficiency, in the small-time sample. The statistically significantly positive, negative, and positive parameters of GDP per capita, its square term, and its cubic term, respectively, would confirm the long-run N-curve and EEKC hypothesis in the models of nonrenewable energy consumption in Equations (7)–(10). On the other hand, the opposite parameters would corroborate the EEKC in Equation (11) of the hydroelectricity model, a clean energy consumption source. Hence, the statistically significant negative, positive and negative parameters of GDP, its square term, and its cubic term, respectively, would confirm the long-run inverted N-curve and the EEKC hypothesis in Equation (11). Moreover, the turning points of N-shaped curves may be estimated by the following formulas suggested by Diao et al. [79]:

(12) GDPC1=(−f2−f22−3∗f1∗f3)3∗f3

(13) GDPC2=(−f2+f22−3∗f1∗f3)3∗f3

**Figure 3:**
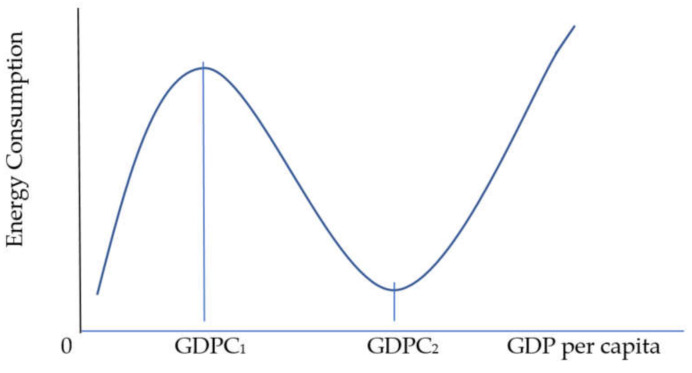
The N-shaped EEKC.

In Equations (12) and (13), *f_1_*, *f_2_*, and *f_3_* are the long-run coefficients of GDPC_t_, GDPC_t_^2^, and GDPC_t_^3^. These long-run coefficients are estimated from Equations (7)–(11). Both formulas are applied to each of Equations (7)–(11) individually to find the first turning point at GDPC_1_ and the second turning point at GDPC_2_ in the case of each energy source. Then, we take exponents of estimated GDPC_1_ and GDPC_2_ to find the GDP per capita because all data are in the natural logarithm. Figure 3 shows the N-shaped EEKC with turning points at GDPC_1_ and GDPC_2_. Before point GDPC_1_, energy consumption is rising with increasing GDP per capita. After GDPC_1_, energy consumption is falling with increasing GDP per capita. After GDPC_2_, energy consumption is growing again with increasing GDP per capita.

(14) $$∆{\mathrm{PEC}}_{t}=a_{31}{\mathrm{ECT}}_{t-1}+\sum_{i=1}^{k1}a_{21i}∆{\mathrm{PEC}}_{t-i}+\sum_{i=0}^{k2}a_{22i}∆{\mathrm{GDPC}}_{t-i}+\sum_{i=0}^{k3}a_{23i}∆{\mathrm{GDPC}}_{t-i}^{2}+\sum_{i=0}^{k4}a_{24i}∆{\mathrm{GDPC}}_{t-i}^{3}+\partial_{1t}$$

(15) $$∆{\mathrm{OC}}_{t}=b_{31}{\mathrm{ECT}}_{t-1}+\sum_{i=1}^{l1}b_{21i}∆{\mathrm{OC}}_{t-i}+\sum_{i=0}^{l2}b_{22i}∆{\mathrm{GDPC}}_{t-i}+\sum_{i=0}^{l3}b_{23i}∆{\mathrm{GDPC}}_{t-i}^{2}+\sum_{i=0}^{l4}b_{24i}∆{\mathrm{GDPC}}_{t-i}^{3}+\partial_{2t}$$

(16) $$∆{\mathrm{NGC}}_{t}=c_{31}{\mathrm{ECT}}_{t-1}\sum_{i=1}^{m1}c_{21i}∆{\mathrm{NGC}}_{t-i}+\sum_{i=0}^{m2}c_{22i}∆{\mathrm{GDPC}}_{t-i}+\sum_{i=0}^{m3}c_{23i}∆{\mathrm{GDPC}}_{t-i}^{2}+\sum_{i=0}^{m4}c_{24i}∆{\mathrm{GDPC}}_{t-i}^{3}+\partial_{3t}$$

(17) $$∆{\mathrm{CC}}_{t}=d_{31}{\mathrm{ECT}}_{t-1}+\sum_{i=1}^{n1}d_{21i}∆{\mathrm{CC}}_{t-i}+\sum_{i=0}^{n2}d_{22i}∆{\mathrm{GDPC}}_{t-i}+\sum_{i=0}^{n3}d_{23i}∆{\mathrm{GDPC}}_{t-i}^{2}+\sum_{i=0}^{n4}d_{24i}∆{\mathrm{GDPC}}_{t-i}^{3}+\partial_{4t}$$

(18) $$∆{\mathrm{HC}}_{t}=e_{31}{\mathrm{ECT}}_{t-1}+\sum_{i=1}^{o1}e_{21i}∆{\mathrm{HC}}_{t-i}+\sum_{i=0}^{o2}e_{22i}∆{\mathrm{GDPC}}_{t-i}+\sum_{i=0}^{o3}e_{23i}∆{\mathrm{GDPC}}_{t-i}^{2}+\sum_{i=0}^{o4}e_{24i}∆{\mathrm{GDPC}}_{t-i}^{3}+\partial_{5t}$$

After cointegration analyses, the statistically significant negative parameters of error correction term (ECT_t-1_) would confirm the short-run relationship in models 14–18 [77]. Then, the short-run EEKC hypothesis may be corroborated with the statistically significant positive, negative, and positive parameters of lag differenced variables of GDPC_t_, its square term, and its cubic term, respectively, in models of nonrenewable energy sources in Equations (14)–(17). The opposing statistical evidence can be claimed to confirm the EEKC in the hydroelectricity model in Equation (18).

## 4. Results

Table 1 shows the descriptive statistics of the utilized variable in the EEKC models. All variables are being used in natural logarithm form, as mentioned in Equations (2)–(6). The standard deviation of all variables shows the significant growth of energy consumption variables and GDP per capita.

Table 2 exposes the Ng-Perron results. The results show that at this level, PEC_t_, OC_t_, NGC_t_, CC_t_, HC_t_, and GDPC_t_ have unit root, and GDPC_t_^2^ and GDPC_t_^3^ are also non-stationary with intercept in analyses. However, GDPC_t_^2^ and GDPC_t_^3^ are level-stationary at 10% and 5% significance level, including intercept and trend in analysis. At first differences, all variables are stationary. Hence, some evidence of mixed order of integration is found. However, cointegration analyses with this problem can be applied, as ARDL is efficient in the mixed order of integration in the model [73].

Table 3 displays the bound test results of models mentioned in Equations (7)–(11). Results show that the cointegration is found in the models of PEC_t_, OC_t_, NGC_t_, and HC_t_ at a 1% level of significance. The bound test could not validate the cointegration in the CC_t_ model. However, the cointegration in the CC_t_ model is alternatively corroborated with estimated negative parameters of ECT_t-1_ in Table 4 [77]. Moreover, diagnostic tests of all estimated models confirm the robustness of the estimated results.

Table 4 shows the estimated parameters of models in Equations (7)–(11). In the long run, the coefficients of LGDPC_t_, LGDPC_t_^2^, and LGDPC_t_^3^ are positive, negative, and positive, respectively, in primary energy, oil, and coal consumption models. Hence, the long-run EEKC hypothesis is corroborated in the N-shaped estimations of these energy proxies. Onafowora and Owoye [72] found an N-shaped relationship between pollutant emissions and economic growth and validated the EKC in the case of Egypt. However, this present study contributes to the literature by providing new evidence of the N-shaped EEKC in the relationship between economic growth and primary energy, oil, and coal consumption. Now, we can calculate the turning points, GPDC_1_ and GDPC_2_, of the N-shaped curve from the formulas mentioned in Equations (12) and (13). In the case of primary and oil consumption models, the square root terms’ results are complex numbers in Equations (12) and (13), which are $\sqrt{f_{2}^{2}-3*f_{1}*f_{3}}$=$\sqrt{-1.6972}$ and $\sqrt{f_{2}^{2}-3*f_{1}*f_{3}}$=$\sqrt{-1.3769}$. Hence, the calculations of turning points are not possible due to the square roots of negative numbers. However, the negative values are too small and would not significantly affect the final outcome of the rest of the formula, mentioned in Equations (12) and (13). If we ignore the square root portion of Equations (12) and (13) with an assumption, then the turning points are approximately located at 23,787 and 25,044 constant Egyptian pounds GDP per capita for primary energy and oil consumption models, which were in 1998 and 2000. Hence, both models show that primary energy and oil consumptions decline negligibly during 1998 and 2000 and continuously rise in the rest of the sample period. We have observed from Figure 1 that oil consumption was more than 50% of total energy consumption until 2001. Hence, total energy (primary energy) and oil consumption rise due to economic growth in most of the sample period. In the coal consumption model, we calculate GDPC_1_ = 13,347 and GDPC_2_ = 27,144 from Equations (12) and (13). Hence, GDPC_1_ is found in 1979, and GDPC_2_ is found in 2005. Hence, economic growth helps to reduce coal consumption during 1979–2005, and accelerates coal consumption in the sample periods 1965–1978 and 2006–2019. Hence, economic growth in recent years is responsible for increasing nonrenewable energy consumption. Therefore, it seems pertinent to float the idea that national policies discourage the consumption of non-renewables. Particularly, these policies should also be suggested at the regional level by territorial governments to discourage urban energy consumption, which might carry a significant proportion of national energy consumption. These policies have also been recommended by the past literature [80,81]. Moreover, all parameters of LGDPC_t_, LGDPC_t_^2^, and LGDPC_t_^3^ are statistically insignificant in the case of natural gas and hydroelectricity consumption. Therefore, the EEKC hypothesis is not corroborated in natural gas and hydroelectricity consumption models. Moreover, none of the monotonic, quadratic, or cubic effects of economic growth on natural consumption and hydroelectricity consumptions could be validated. This conclusion of the non-existence of EEKC is in line with the results of other EKC studies conducted in Egypt, which could not establish any inverted U-shaped or N-shaped relationship between economic growth and pollution emissions [25,28,31,73].

Table 5 shows the estimated short-run coefficients of models in Equations (14)–(18). The short-run relationships are corroborated in all estimated models with the negative and statistically significant parameters of ECT_t-1_ in Equations (14)–(18). Moreover, the coefficients of ΔLGDPC_t_, ΔLGDPC_t_^2^, and ΔLGDPC_t_^3^ are found to be positive, negative, and positive, respectively, in the models of primary energy, oil, and coal consumptions. Hence, the short-run EEKC hypothesis is corroborated in the N-shaped relationship of growth and energy consumption in the case of oil, coal, and primary energy consumption. In the case of natural gas consumption, parameters of ΔLGDPC_t_, ΔLGDPC_t_^2^, and ΔLGDPC_t_^3^ are found to be positive, negative, and statistically insignificant, respectively. Hence, an inverted U-curve EEKC is verified in the natural gas consumption model. The inverted U-shaped EKC in a relationship between pollution emissions and economic growth has been reported by existing studies when conducting time series analysis in Egypt [26,27,30] and performing panel data analysis, including in Egypt [74]. In the hydroelectricity consumption model, an inverted N-shaped relationship is corroborated with negative, positive, and negative parameters of ΔLGDPC_t_, ΔLGDPC_t_^2^, and ΔLGDPC_t_^3^, respectively. Hence, hydroelectricity consumption decreases at the first stage of economic growth, increases at the second stage, and reduces at the third stage. This finding is in line with the finding of an N-shaped relationship between pollution emissions and economic growth in Egypt [72]. We may compare our findings with those of Onafowora and Owoye [72] in that increasing economic growth at the first stage increases pollution, which shows a shift from hydroelectricity or any other renewable to nonrenewable sources of energy. In the second stage, economic growth helps to reduce emissions, which provides evidence of a shift from nonrenewable to renewable sources of energy. In the third stage, emissions increase with economic growth, which again shows a shift from renewable to nonrenewable sources. Hence, the short-run EEKC is verified as hydroelectricity consumption is a renewable energy source. On the whole, the short-run EEKC is validated in the case of all estimated models.

In summary, the long run EEKC is corroborated in primary energy, oil, and coal consumption in Egypt. Hence, economic growth is responsible for increasing oil, coal, and primary energy consumption in most sample years, all nonrenewable. Therefore, increasing such energy consumption types is responsible for environmental degradation. On the other hand, economic growth could not help to increase hydroelectricity consumption. Moreover, the short-run EEKC is confirmed in all the investigated models. Though the present study did not include the effects of globalization and economic openness on energy consumption behavior, the literature has highlighted the role of trade in shaping the EKC in a relationship between pollutant emissions and economic growth [1,36]. Furthermore, the previous environmental literature has corroborated that trade and FDI have helped to reduce pollutant emissions in Egypt [25,26]. Hence, the openness of the economy has contributed to shaping of the EKC in Egypt.

## 5. Conclusions

The testing of the EKC between economic growth and pollutant emissions is abundant in the environmental literature. Nevertheless, EEKC hypothesis testing in the relationship between energy use and economic growth is limited globally and has not been conducted in Egyptian literature. Hence, we investigated the EEKC in the N-shaped relationship between growth and different renewable and nonrenewable proxies of energy consumption in Egypt from 1965–2019. For this purpose, we apply the ARDL cointegration technique. The long-run results corroborate the N-curve in the case of primary energy consumption, oil consumption, and coal consumption models. Hence, the long-run EEKC is verified in these energy consumption proxies. Both oil and primary consumption reduced negligibly in 1998 and 2000 and had a positive relationship with economic growth in the rest of the sample period. Hence, primary energy and oil consumption increase along with economic growth in most of the sample period. However, we calculated both turning points of the N-curve between coal consumption and economic growth. We found the first turning point at 13,347 and the second at 27,144 located at years 1979 and 2005, respectively. Therefore, the economic growth reduced the coal consumption during 1979–2005 and is responsible for increasing coal consumption in the rest of the sample periods 1965–1978 and 2006–2019. On the other hand, the long-run EEKC is invalid in the case of hydroelectricity and natural gas consumption. Hence, economic growth in Egypt has environmental consequences in most of the sample period. We find the N-shaped relationship in primary energy, oil, and coal consumption models in the short-run results. In addition, we find a short-run inverted U-curve in the natural gas consumption model. Moreover, a short-run inverted N-curve is corroborated in hydroelectricity, which is a clean energy source. Hence, the short-run EEKC has been found in all investigated energy sources. Previous literature has incorporated and corroborated the role of the openness of the economy in shaping the EKC in the relationship between pollutant emissions and economic growth in Egypt [25,26]. Hence, a future study may consider the role of free trade and other open measures in the relationship between economic growth and energy consumption when investigating the EEKC.

## Figures and Tables

**Table 1:** Descriptive Statistics.

	OC_t_	NGC_t_	CC_t_	PEC_t_	HC_t_	GDPC_t_
Mean	−0.224187	−1.898542	−3.575728	0.244363	−2.411493	9.861380
Median	−0.025270	−1.078036	−3.498677	0.364126	−2.281486	9.908434
Maximum	0.567938	0.763329	−2.406410	1.365439	−1.941707	10.55649
Minimum	−1.612627	−6.378393	−4.467861	−1.341031	−4.055660	9.082881
Std. Dev.	0.628077	2.467149	0.417401	0.843266	0.510672	0.470406
Observations	55	55	55	55	55	55

**Table 2:** Unit root results.

Variable	Intercept				Intercept and Trend			
PEC_t_	−4.2658	−1.2638	0.2963	6.0270	−3.9478	−1.2643	0.3203	21.4005
OC_t_	0.5895	0.6476	1.0985	75.7772	−2.9230	−1.0404	0.3560	26.7822
NGC_t_	0.8080	1.0480	1.2969	107.9720	−2.3539	−0.9181	0.3900	31.7657
CC_t_	−2.6936	−0.7081	0.2629	7.7003	−8.3180	−1.8994	0.2284	11.3901
HC_t_	−0.8039	−0.4699	0.5846	19.8411	−5.0146	−1.4586	0.2909	17.5758
GDPC_t_	0.9867	0.8374	0.8486	51.9301	−9.0978	−2.0891	0.2296	10.1875
GDPC_t_^2^	1.0413	0.8770	0.8422	51.7753	−15.0243 *	−2.7179 *	0.1809 *	6.2016 *
GDPC_t_^3^	1.0959	0.9141	0.8341	51.4475	−18.1113 **	−2.9987 **	0.1656 **	5.0958 **
ΔPEC_t_	−26.0071 ***	−3.5823 ***	0.1377 ***	1.0198 ***	−25.9332 ***	−3.5905 ***	0.1385 ***	3.5789 ***
ΔOC_t_	−26.4147 ***	−3.6155 ***	0.1369 ***	0.9885 ***	−26.2858 ***	−3.6236 ***	0.1379 ***	3.4769 ***
ΔNGC_t_	−35.7491 ***	−4.2218 ***	0.1181 ***	0.7030 ***	−140.6270 ***	−8.3848 ***	0.0596 ***	0.6496 ***
ΔCC_t_	−4.3428	−1.4666	0.3376	5.6538	−25.7829 ***	−3.5668 ***	0.1383 ***	3.6748 ***
ΔHC_t_	−16.7487 ***	−2.8835 ***	0.1722 ***	1.5016 ***	−20.2622 **	−3.1806 **	0.1569 **	4.5127 **
ΔGDPC_t_	−24.3503 ***	−3.4847 ***	0.143 1 ***	1.0217 ***	−24.3679 ***	−3.4874 ***	0.1431 ***	3.7588 ***
ΔGDPC_t_^2^	−25.9124 ***	−3.5908 ***	0.1389 ***	0.9739 ***	−25.6068 ***	−3.5743 ***	0.1396 ***	3.5819 ***
ΔGDPC_t_^3^	−27.5211 ***	−3.6954 ***	0.1343 ***	0.1343 ***	−27.1362 ***	−3.6787 ***	0.1356 ***	3.3862 ***

Note: ***, ** and * show stationarity at 1%, 5%, and 10% significant levels.

**Table 3:** Bound testing.

Model	F-Stat.	Hetero.	Serial Correlation	Normality	Functional Form
PEC_t_	4.6340	0.9459 (0.3354)	1.6229 (0.2087)	26.5508 (0.0000)	2.3367 (0.1341)
OC_t_	7.7437	2.0329 (0.1600)	1.8552 (0.1795)	34.215 (0.0000)	1.9615 (0.1520)
NGC_t_	7.5839	16.1084 (0.0000)	1.3060 (0.2806)	106.3483 (0.0000)	0.0027 (0.9589)
CC_t_	1.7022	1.0385 (0.3130)	0.6389 (0.4282)	1.4937 (0.4739)	0.1413 (0.7088)
HC_t_	7.0509	0.5800 (0.5637)	0.6694 (0.5174)	8.8042 (0.0123)	2.5999 (0.1142)
Critical F-statistics					
At 1 percent	4.0934–4.9199				
At 5 percent	3.0836–3.8155				
At 10 percent	2.6175–3.2969				

**Table 4:** Long run results.

Dependent Variable	Independent Variable	Coefficient	Std. Error	*t*-Statistic	Prob.
PEC_t_	LGDPC_t_	112.0502	47.7788	2.3452	0.0233
	LGDPC_t_^2^	−10.9647	4.8552	−2.2584	0.0286
	LGDPC_t_^3^	0.3627	0.1642	2.2083	0.0321
	Intercept	−386.1950	156.5130	−2.4675	0.0173
OC_t_	LGDPC_t_	188.7147	88.1676	2.1404	0.0373
	LGDPC_t_^2^	−18.4347	8.9762	−2.0537	0.0454
	LGDPC_t_^3^	0.6027	0.3042	1.9812	0.0532
	Intercept	−646.3510	288.2987	−2.2420	0.0295
NGC_t_	LGDPC_t_	317.8377	221.3655	1.4358	0.1574
	LGDPC_t_^2^	−29.1443	22.3645	−1.3032	0.1986
	LGDPC_t_^3^	0.8958	0.7521	1.1909	0.2394
	Intercept	−1160.3700	729.3086	−1.5911	0.1180
CC_t_	LGDPC_t_	1700.2550	1005.4270	1.6911	0.0974
	LGDPC_t_^2^	−172.7690	102.4808	−1.6859	0.0985
	LGDPC_t_^3^	5.8443	3.4770	1.6808	0.0994
	Intercept	−5573.6000	3283.2480	−1.69759	0.0962
HC_t_	LGDPC_t_	−430.89	442.13	−0.9746	0.3351
	LGDPC_t_^2^	43.2323	44.5783	0.9698	0.3374
	LGDPC_t_^3^	−1.4422	1.4968	−0.9635	0.3406
	Intercept	1425.3810	1460.2370	0.9761	0.3343

**Table 5:** Short run results.

Dependent Variable	Independent Variable	Coefficient	Std. Error	t-Statistic	Prob.
ΔPEC_t_	ΔPEC_t-1_	−0.2232	0.1081	−2.0641	0.0445
	ΔLGDPC_t_	88.5923	32.5737	2.7198	0.0091
	ΔLGDPC_t_^2^	−8.6692	3.3243	−2.6079	0.0122
	ΔLGDPC_t_^3^	0.2868	0.1128	2.5421	0.0144
	ECT_t−1_	−0.7907	0.1274	−6.2055	0.0000
ΔOC_t_	ΔLGDPC_t_	104.8086	44.8486	2.3369	0.0236
	ΔLGDPC_t_^2^	−10.2383	4.5717	−2.2395	0.0297
	ΔLGDPC_t_^3^	0.3348	0.1552	2.1572	0.0359
	ECT_t−1_	−0.5554	0.1067	−5.2033	0.0000
ΔNGC_t_	ΔLGDPC_t_	215.6292	111.8583	1.9277	0.0597
	ΔLGDPC_t_^2^	−19.7723	11.4202	−1.7313	0.0897
	ΔLGDPC_t_^3^	0.6077	0.3892	1.5614	0.1249
	ECT_t−1_	−0.6784	0.2956	−2.2951	0.0260
ΔCC_t_	ΔLGDPC_t_	389.3343	173.3717	2.2457	0.0295
	ΔLGDPC_t_^2^	−42.3082	18.3057	−2.3112	0.0253
	ΔLGDPC_t_^3^	1.5236	0.6453	2.3610	0.0224
	ECT_t−1_	−0.2290	0.1140	−2.0091	0.0503
ΔHC_t_	ΔHC_t−1_	0.3983	0.1356	2.9378	0.0052
	ΔLGDPC_t_	−535.0280	233.1376	−2.2949	0.0266
	ΔLGDPC_t_^2^	54.6496	23.8973	2.2869	0.0271
	ΔLGDPC_t_^3^	−1.8540	0.8150	−2.2748	0.0278
	ECT_t−1_	−0.1319	0.0548	−2.4068	0.0204

## Data Availability

The data are publicly available [15,58].
